# Anti-Inflammatory and Antiatopic Effects of *Rorippa cantoniensis* (Lour.) Ohwi in RAW 264.7 and HaCaT Cells

**DOI:** 10.3390/molecules28145463

**Published:** 2023-07-17

**Authors:** Min-Jin Kim, Buyng Su Hwang, Yong Hwang, Yong Tae Jeong, Dae Won Jeong, Young Taek Oh

**Affiliations:** 1 Nakdonggang National Institute of Biological Resources, 137, Donam 2-gil, Sangju-si 37242, Republic of Korea; hwang1531@nnibr.re.kr (B.S.H.); hdragon@nnibr.re.kr (Y.H.); ytjeong@nnibr.re.kr (Y.T.J.); jdo1225@nnibr.re.kr (D.W.J.); 2 Department of Life Sciences, Kyungpook National University (KNU), 80 Daehak-ro, Buk-gu, Daegu 41566, Republic of Korea

**Keywords:** inflammatory, antiatopic activity, MAPK, NF-κB, STAT1, *Rorippa cantoniensis* (Lour.) Ohwi

## Abstract

This study evaluated the effects of *Rorippa cantoniensis* (Lour.) ohwi extract (RCE) on factors associated with inflammation-related skin lesions in RAW 264.7 and HaCaT cells. RCE inhibited the levels of proinflammatory mediators and cytokines such as nitric oxide (NO), prostaglandin E2 (PGE_2_), interleukin (IL)-6, and tumor necrosis factor (TNF)-α in lipopolysaccharide (LPS)-stimulated RAW 264.7 cells. In addition, RCE significantly inhibited the expression of chemokines and cytokines such as MDC/CCL22, TARC/CCL17, RANTES/CCL5, CTSS, IL-6, IL-1β, and TNF-α in HaCaT cells costimulated by TNF-α and interferon (IFN)-γ in a concentration-dependent manner. These results suggest that RCE attenuated the TNF-α- and IFN-γ-induced release of proinflammatory chemokines and cytokines probably by suppressing the activation of MAPK (JNK and p38), NF-κB, and STAT1 signaling. Moreover, RCE significantly increased the expression of skin components such as hyaluronic acid and aquaporin, which play important roles in the physical and chemical barriers of the skin. These results suggest that RCE has significant anti-inflammatory and antiatopic activities, which may be beneficial for the topical treatment of inflammatory skin disorders.

## 1. Introduction

Atopic dermatitis (AD)-like skin diseases are the most common chronic inflammatory skin disorders caused by innate and adaptive immune responses based on genetic, seasonal, and environmental factors [1]. AD is caused by complex interactions between extrinsic and intrinsic factors. The main risk factors for AD are not yet known; however, it is well established that immune system dysfunction and environmental factors, such as mite dust, food-allergen exposure, and smoking, impair the skin barrier and exacerbate immunoglobulin E (IgE)-mediated sensitization, severe skin inflammation, and immune responses [2,3].

For example, in inflamed skin, cytokines and chemokines are produced by resident cells, including keratinocytes, mast cells, macrophages, and Langerhans cells, and by infiltrated cells, including lymphocytes and neutrophils [4]. Among these cells, AD is closely related to keratinocytes and immune cells. Macrophages play a central role in the management of many pathological immune phenomena such as the overproduction of inflammatory mediators. Various mediators, such as nitric oxide (NO), prostaglandins E_2_ (PGE_2_), and inflammatory cytokines, including interleukin (IL)-6, IL-1β, tumor necrosis factor (TNF)-α, and others induced by macrophages, have important roles in inflammatory diseases [5]. Keratinocytes, which exist in more than 80% of the human stratum corneum, recognize these as antigens when stimulated from outside the skin. Keratinocytes contribute to the structural integrity of the skin and inflammatory responses by producing proinflammatory cytokines and chemokines. RAW 264.7 macrophages and HaCaT keratinocytes are the representative cell lines used for anti-inflammatory activity studies.

Stimulation of keratinocytes with tumor necrosis factor (TNF)-α and interferon-γ (IFN-γ) leads to the expression of proinflammatory cytokines, chemokines, and adhesion molecules, which play an important role in the migration of inflammatory cells to the site of inflammation on the skin [6]. Keratinocyte-derived cytokines and chemokines, such as interleukin (IL)-6, IL-1β, TNF-α, macrophage-derived chemokine (MDC/CCL22), thymus- and activation-regulated chemokine (TARC/CCL17), regulated on activation, normal T-cell expressed and secreted (RANTES), thymic stromal lymphopoietin (TSLP), Cathepsin S (CTSS), and monocyte chemoattractant protein-1 (MCP-1) [7,8]. High concentrations of TARC/CCL17, MDC/CCL22, TSLP, and RANTES have been detected in patients with AD [9]. These biomarkers are also closely associated with atopic disease [10].

The combination of TNF-α and IFN-γ activates several intracellular pathways, including NF-κB pathways [11]. NF-κB pathways have been shown to regulate chemokine and cytokine production in keratinocyte cells. They play a significant role in immune responses and regulate inflammatory signaling [12].

Other studies have reported that the signal transducer and activator of transcription 1 (STAT1) and mitogen-activated protein kinase (MAPK) play critical roles in chemokine production mediated by tumor necrosis factor-alpha (TNF-α) and/or interferon-gamma (IFN-α) in keratinocytes [13]. Chronic skin diseases in patients with AD are caused by decreased expression of antimicrobial peptides and abnormal skin components (including involucrin, loricrin, filaggrin, hyaluronic acid, and aquaporins) [14].

Currently, topical ointments and oral medications, such as corticosteroids (e.g., glucocorticoids), calcineurin inhibitors (e.g., tacrolimus), and antihistamines, are commonly used to treat AD [15]. Treatment drugs, such as corticosteroids or calcineurin inhibitors, can temporarily relieve the symptoms of AD by reducing inflammation; however, long-term use of drugs may cause side effects such as local cutaneous atrophy, stinging, itching, telangiectasia, and burning sensation [16]. Therefore, to minimize the adverse effects of long-term use of AD drugs, it is necessary to develop new alternative drugs derived from natural resources with reasonable prices and fewer side effects. Hence, the development of alternative therapeutic agents for the treatment of AD, especially bioactive compounds extracted from natural resources, has received increasing interest.

*Rorippa cantoniensis* (Lour.) Ohwi (RC), an annual plant (10–40 cm in size) belonging to the Brassicaceae family, commonly grows in wet places along rivers or near rice fields. In previous studies, it was confirmed that the RC extract had excellent anti-inflammatory efficacy in RAW 264.7 cells [17]. However, the mechanisms of RC in atopic dermatitis in terms of its therapeutic effect are not clear.

This study aimed to evaluate whether *Rorippa cantoniensis* (Lour.) Ohwi extract (RCE) could be utilized as a natural therapeutic agent against AD. We investigated the anti-inflammatory and antiatopic effects of RCE in vitro using RAW264.7 macrophages and HaCaT human keratinocytes.

## 2. Results

### 2.1. Cytotoxicity of RCE in RAW 264.7 and HaCaT Cells

**Figure 1:**
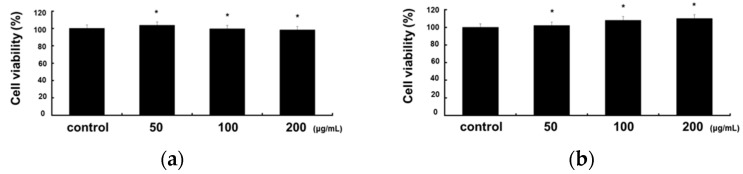
Effects of RCE on cell viability in RAW 264.7 cells and HaCaT Cells. (**a**) RAW 264.7 (2.0 × 10^5^ cells/mL) and (**b**) HaCaT Cells (2.0 × 10^5^ cells/mL) were preincubated for 18 h in 96-well plates, the cells were treated with various concentrations of RCE (50, 100, and 200 μg/mL) for 48 h. Cell viability was determined using the MTT assay. All data are presented as the mean ± S.D. of triplicate experiments. * *p* < 0.05 vs. nontreated group.

To investigate whether RCE exhibits cytotoxic effects, we first performed MTT assays to determine whether RCE was cytotoxic to RAW 264.7 and HaCaT cells. The viability of RAW 264.7 and HaCaT cells was measured 48 h after treatment with 50, 100, or 200 μg/mL RCE. As shown in Figure 1a,b, RCE had no significant effect on cell viability. Therefore, in the following experiments, RAW 264.7 and HaCaT cells were treated with 50, 100, and 200 μg/mL RCE.

### 2.2. Inhibitory Effects of RCE on LPS-Induced Inflammatory Mediator and Proinflammatory Cytokines in RAW 264.7 Cells

**Figure 2:**
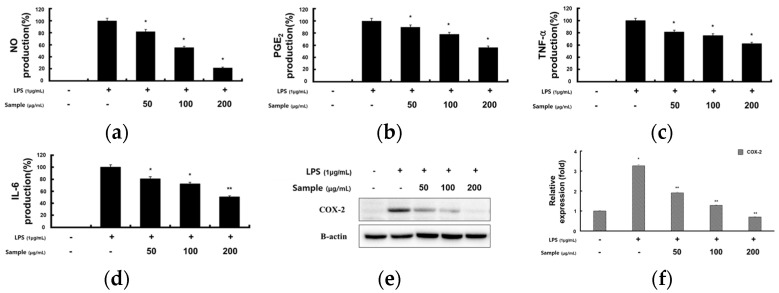
Effects of RCE on LPS-stimulated production of proinflammatory mediators in RAW 264.7 cells. RAW 264.7 cells (2.0 × 10^5^ cells/mL) were preincubated for 18 h in 24-well plates and then stimulated with LPS (1 μg/mL) and RCE (50, 100, and 200 μg/mL) for 24 h. (**a**) NO production was measured using the Griess reagent. (**b**) PGE_2_, (**c**) TNF-α, and (**d**) IL-6 production was determined by ELISA kit. (**e**) The expression levels of COX-2 were examined by Western blotting. (**f**) Protein level of COX-2. All data are presented as the mean ± S.D. of triplicate experiments. * *p* < 0.05 and ** *p* < 0.01 vs. group treated with LPS alone.

Macrophages produce inflammatory mediators, including NO, PGE_2_, and proinflammatory cytokines, which attract immune cells to the site of infection and activate cells to eliminate them [18]. To investigate the effects of RCE on LPS-induced NO and PGE_2_ production in RAW 264.7 cells, the culture media were harvested and the NO and PGE_2_ levels were measured using Griess reagent and ELISA, respectively. LPS-treated cells showed increased NO and PGE_2_ production compared to normal cells. RCE treatment also significantly inhibited NO and PGE_2_ production (Figure 2a,b). Furthermore, to investigate the RCE-mediated production of proinflammatory cytokines, we examined whether RCE can affect the secretion of TNF-α and IL-6 in LPS-stimulated RAW 264.7 cells. RCE significantly inhibited the expression of proinflammatory cytokines (TNF-α, and IL-6) in LPS-stimulated RAW 264.7 cells (Figure 2c,d).

Since RCE was found to inhibit PGE_2_ production, we investigated whether these inhibitory effects were related to COX-2 modulation using Western blotting. As shown in Figure 2e, COX-2 protein expression was dramatically increased following treatment with LPS alone compared to the control group, whereas pretreatment with RCE decreased the protein levels of COX-2 in a dose-dependent manner. This reduction in COX-2 protein expression was consistent with the observed inhibition of PGE_2_.

### 2.3. Inhibitory Effects of RCE on TNF-α/IFN-γ-Induced Proinflammatory Cytokines and Chemokines in HaCaT Cells

Skin immune diseases such as AD and allergies are caused by excessive skin inflammation. Since keratinocytes activated by various stimuli play an important role in the inflammatory immune response [19], we examined the anti-inflammatory effects of RCE on HaCaT cells under TNF-α and IFN-γ-induced inflammatory conditions.

**Figure 3:**
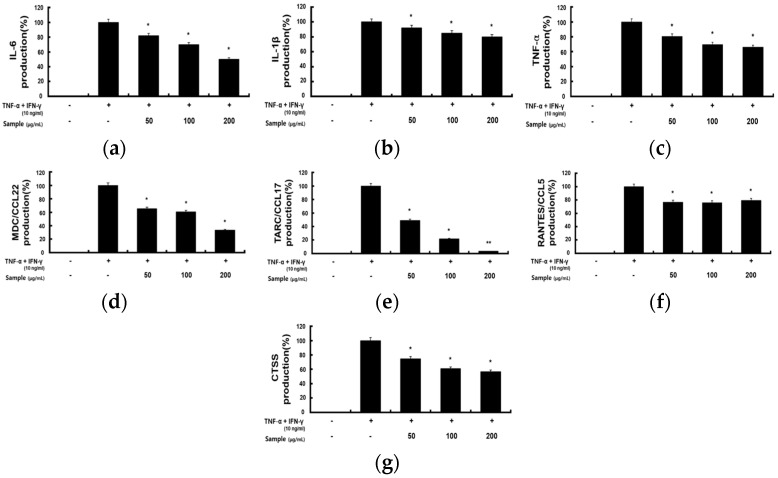
Effects of RCE on TNF-α and IFN-γ induced production of proinflammatory cytokines and chemokines in HaCaT cells. HaCaT cells (2.0 × 10^5^ cells/mL) were preincubated for 18 h in 24 well plates and then stimulated with TNF-α and IFN-γ (each 10 ng/mL) and RCE (50, 100, and 200 μg/mL) for 24 h. Levels of (**a**) IL-6, (**b**) IL-1β, (**c**) TNF-α, (**d**) MDC/CCL22, (**e**) TARC/CCL17, (**f**) RANTES/CCL5, and (**g**) CTSS were measured using the ELISA kit. All data are presented as the mean ± S.D. of triplicate experiments. * *p* < 0.05 and ** *p* < 0.01 vs. group treated with TNF-α and IFN-γ alone.

To investigate the inhibitory effect of RCE on proinflammatory chemokines and cytokines, HaCaT cells pretreated with RCE were stimulated with TNF-α and IFN-γ. RCE significantly reduced the production of cytokines (IL-6, IL-1β, and TNF-α) and chemokines (MDC/CCL22, TARC/CCL17, RANTES/CCL5, and CTSS) in HaCaT cells costimulated by TNF-α and IFN-γ in a concentration-dependent manner (Figure 3a–g). Thus, RCE suppressed the expression of cytokines and chemokines in TNF-α and IFN-γ-induced HaCaT cells.

### 2.4. Inhibitory Effects of RCE on the Expression of NF-κB and STAT1 Signaling Pathway in TNF-α/IFN-γ-Induced HaCaT Cells

**Figure 4:**
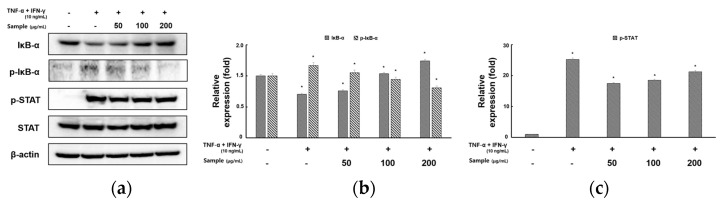
Effects of RCE on TNF-α and IFN-γ induced NF-κB and STAT1 signaling pathway in HaCaT cells. The cells were pretreated with 50, 100, and 200 μg/mL RCE for 2 h, followed by exposure to TNF-α and IFN-γ (10 ng/mL each) for 30 min. Cell extracts were prepared and NF-κB and STAT1 activation was analyzed by Western blotting using specific antibodies. (**a**) Results of Western blotting and protein levels of (**b**) phospho-IκB-α and IκB-α and (**c**) phosphor-STAT1. Equal protein loadings were confirmed by measuring phospho-IκB-α, IκB-α, STAT1, and β-actin expression. Protein levels were quantified using the Image Lab software (version 5.2.1). All data are presented as the mean ± S.D. of triplicate experiments. * *p* < 0.05 vs. TNF-α- and IFN-γ-stimulated cells.

TNF-α- and IFN-γ-induce the release of chemokines and cytokines by activating NF-κB in keratinocytes [20,21]. To examine whether the influence of RCE could downregulate MDC expression via the inhibition of NF-κB activation, we evaluated the effect of RCE on NF-κB activity. RCE treatment suppressed TNF-α-induced and IFN-γ-induced IκB-α phosphorylation and IκB-α degradation (Figure 4a,b). STAT-1 has been implicated in AD-like skin-lesion-related signaling in TNF-α- and IFN-γ-activated cells [22]. We evaluated the inhibitory effect of RCE on TNF-α- and IFN-γ-induced STAT1 activation by Western blotting. TNF-α- and IFN-γ activated phosphorylation of STAT1 but the effect was reduced by RCE treatment (Figure 4a,c).

### 2.5. Inhibitory Effects of RCE on the Expression of MAPK Signaling Pathway in TNF-α/IFN-γ-Induced HaCaT Cells

**Figure 5:**
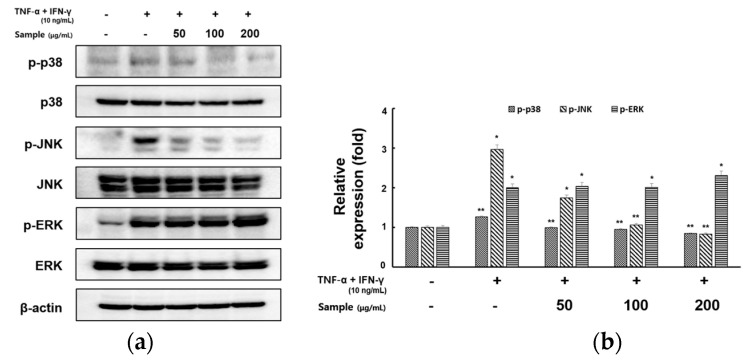
Effects of RCE on TNF-α and IFN-γ induced MAPK signaling pathway in HaCaT cells. The cells were pretreated with 50, 100, and 200 μg/mL RCE for 2 h, followed by exposure to TNF-α and IFN-γ (10 ng/mL each) for 30 min. Cell extracts were prepared and MAPK activation was analyzed by Western blotting using specific antibodies. (**a**) Results of Western blotting and (**b**) protein levels of MAPKs. Equal protein loadings were confirmed by measuring phospho-ERK, ERK, phospho-JNK, JNK, phospho-p38, p38, and actin expression. Protein levels were quantified using the Image Lab software (version 5.2.1). All data are presented as the mean ± S.D. of triplicate experiments. * *p* < 0.05 and ** *p* < 0.01 vs. TNF-α- and IFN-γ-stimulated cells.

MAPK is an important signaling factor in keratinocyte activation [23]. We next evaluated the inhibitory effect of RCE on TNF-α- and IFN-γ-induced MAPK activation by Western blotting. As shown in Figure 5a,b, pretreatment with RCE resulted in the suppression of p38 and JNK in a concentration-dependent manner, whereas the levels of phosphorylated ERK were not affected. 

**Figure 6:**
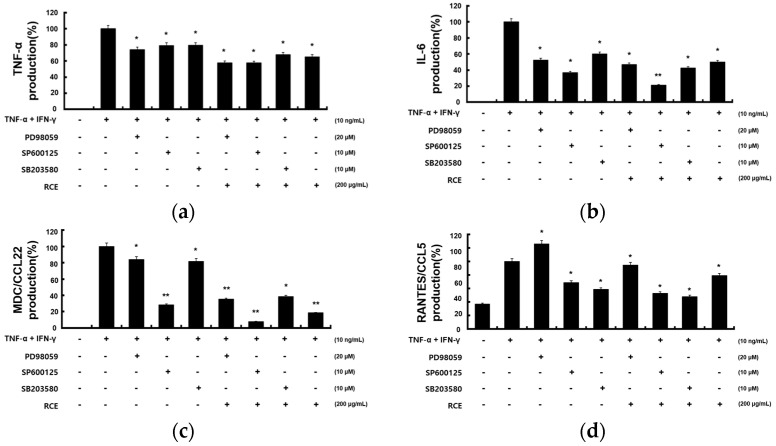
Effects of MAPK inhibitors on the expression of pro-inflammatory cytokines and chemokines. HaCaT cells were pretreated with RCE (200 μg/mL), SP600235 (10 μM), SB203580 (10 μM), and PD98059 (20 μM) for 2 h before culturing with 10 ng/mL of TNF-α and IFN-γ. (**a**) TNF-α, (**b**) IL-6, (**c**) MDC/CCL22, and (**d**) TARC/CCL17 levels were measured using ELISA kits. All data are presented as the mean ± S.D. of triplicate experiments. * *p* < 0.05 and ** *p* < 0.01 vs. TNF-α- and IFN-γ-stimulated cells.

Furthermore, we aimed to identify the precise MAPK signaling pathway involved in the RCE inhibition of TNF-α- and IFN-γ-induced production of proinflammatory cytokines (TNF-α, IL-6) and chemokines (MDC/CCL22, RANTES/CCL5) in HaCaT cells using specific kinase inhibitors for ERK(PD98059), JNK(SP600125), and p38(SB203580). As shown in Figure 6a–d, treatment with RCE and inhibitors significantly suppressed TNF-α- and IFN-γ-induced expression of proinflammatory cytokines and chemokines.

### 2.6. Effects of RCE on Other Molecules in HaCaT Cells

**Figure 7:**
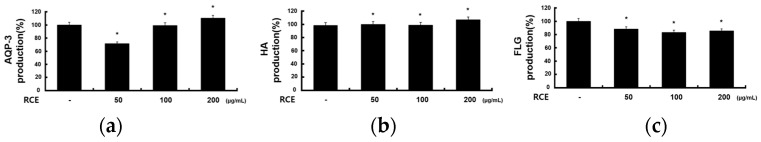
Effects of RCE on TNF-α and IFN-γ induced production of the physical barrier function and skin hydration in HaCaT cells. HaCaT cells were pretreated with RCE (200 μg/mL) for 24 h. (**a**) Aquaporin (AQP3), (**b**) hyaluronic acid (HA), and (**c**) filaggrin levels were measured using ELISA. All data are presented as the mean ± S.D. of triplicate experiments. * *p* < 0.05 vs. RCE not treated group.

In addition to evaluating the inflammatory mediators and signaling molecules involved in AD, we investigated the effects of RCE on factors related to the physical structure of HaCaT cells. To investigate the effects of RCE on skin components, such as hyaluronic acid, aquaporin, and filaggrin, HaCaT cells were treated with RCE. As shown in Figure 7, RCE increased the production of hyaluronic acid and aquaporin in a dose-dependent manner but did not affect the production of filaggrin.

### 2.7. UPLC-MS/MS Analysis of RCE

**Figure 8:**
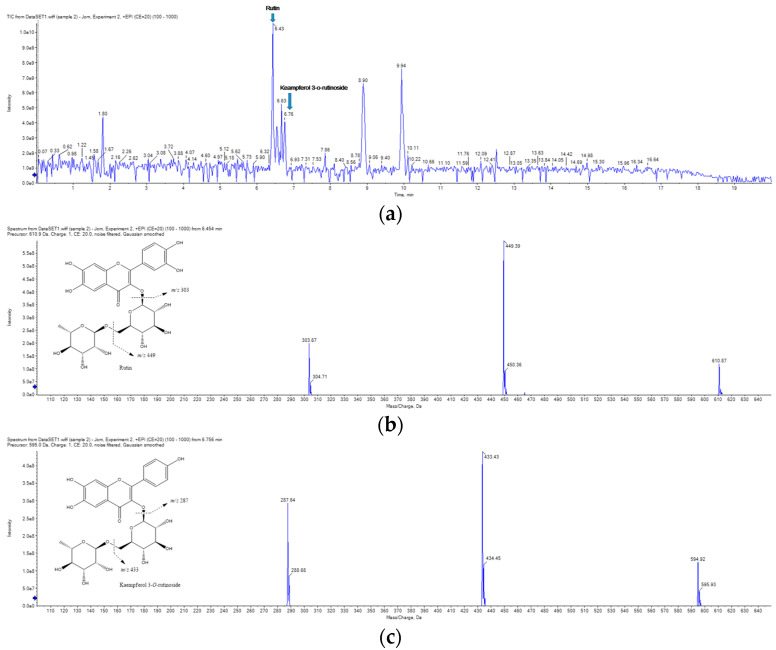
UPLC-MS/MS analysis of RCE. TIC (total ionization chromatogram) of the (**a**) RCE, mass spectra of (**b**) rutin fragmentation, and (**c**) kaempferol 3-*O*-rutinoside fragmentation.

The main components of the RCE were determined using UPLC-MS/MS Figure 8. The main chemical constituents (rutin, kaempferol 3-*O*-rutinoside) were identified by reference literature and mass fragments [24].

## 3. Materials and Methods

### 3.1. Chemicals and Reagents

LPS, phosphate-buffered saline (PBS), dimethyl sulfoxide (DMSO), 3-(4,5-dimethylthiazol-2-yl)-2,5-diphenyltetrazolium bromide (MTT), and RIPA lysis buffer were purchased from Sigma-Aldrich (St. Louis, MO, USA). Fetal bovine serum (FBS) and Dulbecco’s modified Eagle medium (DMEM) were purchased from Invitrogen (Grand Island, NY, USA). Recombinant human TNF-α and IFN-γ were purchased from BioLegend (San Diego, CA, USA). Enzyme-linked immunosorbent assay (ELISA) kits for the analyses of TNF-α, IL-6, and PGE_2_ were obtained from BD Biosciences (San Diego, CA, USA) and R&D Systems, Inc. (St. Louis, MO, USA). Antiphosphorylated IκB-α (anti-p-IκB-α), anti-NF-κB, anti-JNK, anti-p38, anti-ERK1/2, antiphosphorylated JNK (anti-p-JNK), antiphosphorylated p38 (anti-p-p38), and antiphosphorylated ERK1/2 (anti-p-ERK1/2) mouse or rabbit antibodies were purchased from Cell Signaling Technology (Beverly, MA, USA) or Santa Cruz Biotechnology Inc. (Dallas, TX, USA). All other reagents were obtained from Sigma-Aldrich.

### 3.2. Plant Material

The Plant (*Rorippa cantoniensis* (Lour.) ohwi) sample used in this experiment was directly collected from individuals with recognition traits in Yoseong-ri, Mungyeong-eup, Mungyeong-si, Gyeongsangbuk-do, Republic of Korea (April 2017), dried and crushed, and this specimen is stored in the storage of the National Nakdonggang Biological Resources Center (NNIBRVP-60101). Identification of plants was classified according to the ‘Korea Biodiversity Information System’. *Rorippa cantoniensis* (Lour.) Ohwi (RC) were collected from Mungyeong, Gyeongsangbuk-do. Plant samples were freeze-dried and ground into a fine powder using a blender. The dried powder (50 g) was added to 70% EtOH (70% EtOH, 1 L) at room temperature for 2 d to prepare the extract. The yield of 70% ethanol extract was 11.2%.

### 3.3. Cell Culture

RAW 264.7 murine macrophages and HaCaT cells (human keratinocytes) were purchased from the Korean Cell Line Bank (Seoul, Republic of Korea). These cells were maintained at subconfluence in a 5% CO_2_ humidified atmosphere at 37 °C and the medium was changed every two or three days during incubation. The medium used for the routine subculture was Dulbecco’s modified Eagle medium (DMEM) supplemented with 10% fetal bovine serum (FBS), penicillin (100 units/mL), and streptomycin (100 μg/mL).

### 3.4. Cell Viability Assay

The effect of RC extract on the viability of RAW 264.7 and HaCaT cells was assessed using the MTT assay. RAW 264.7 cells and HaCaT cells at a density of 2.0 × 10^5^ cells/well were seeded into a 96-well plate and treated with various concentrations of RCE (50, 100, and 200 μg/mL), and incubated for 24 h. Following incubation, MTT solution was added to each well, and the plates were further incubated for 4 h in the dark. The supernatant of each well was discarded, and the crystallized formazan was dissolved in dimethyl sulfoxide (DMSO) (200 L/well). The absorbance was measured at 540 nm. The percentage of cells exhibiting cytotoxicity was determined relative to the control group.

### 3.5. Nitric Oxide (NO) Determination

The nitrite concentration in the medium was measured as an indicator of NO production according to the Griess reaction method. In brief, RAW 264.7 cells (1.8 × 10^5^ cells/mL) were plated in 24-well plates, incubated for 24 h, pretreated with the indicated concentrations of the RCE for 2 h, and then challenged with LPS (1 μg/mL) for an additional 18 h. Equal volumes of cultured medium and Griess reagent (1% sulfanilamide and 0.1% N-(1-naphthyl)-ethylene diaminedihydrochloride in 5% phosphoric acid) were mixed at room temperature for 10 min, and absorbance was measured at 540 nm.

### 3.6. Enzyme-Linked Immunosorbent Assay (ELISA)

RAW 264.7 cells (2.0 × 10^5^ cells/mL) were cultured in 24-well plates in the presence of various concentrations (50, 100, and 200 μg/mL) of RCE and LPS (1 μg/mL) and incubated for 24 h. The cell culture medium was centrifuged at 13,000 rpm for 10 min and the supernatants were collected for PGE_2_, IL-6, and TNF-α analyses performed using an ELISA kit according to the manufacturer’s instructions. HaCaT cells (2.0 × 10^5^ cells/mL) were cultured in 24-well plates in the presence of various concentrations (50, 100, and 200 μg/mL) of RCE and TNF-α/IFN-γ (each 10 ng/mL) and incubated for 24 h. The cell culture medium was centrifuged at 13,000 rpm for 10 min and the supernatants were collected for the analysis of TARC/CCL17, MDC/CCL22, RANTES/CCL5, Cathepsin S(CTSS), IL-6, IL-1β, and TNF-α, performed using an ELISA kit according to the manufacturer’s instructions.

### 3.7. Western Blot Analysis

Protein expression of MAPKs, NF-κB, STAT1, and β-actin was detected by Western blotting. RAW 264.7 cells were pretreated with various doses of RCE (50, 100, and 200 μg/mL) for 2 h, followed by treatment with LPS (1 µg/mL) for 24 h. HaCaT cells were pretreated with various doses of RCE (50, 100, and 200 μg/mL) for 2 h followed by treatment with TNF-α/IFN-γ (10 ng/mL) for 30 min. The cells were allowed to lyse in lysis buffer (RIPA buffer, 1% Nonidet P-40, 1% protease inhibitor cocktail) for 1 h, collected in microtubes, and then centrifuged at 15,000 rpm for 15 min at 4 °C. The supernatants were transferred to new microtubes. The protein content of the cell lysates was determined using the Bradford reagent (Bio-Rad, Hercules, CA, USA) with bovine serum albumin (BSA) as a standard. After heating at 70 °C for 10 min, equal amounts of the cell lysates were separated using 4–12% Bis-Tris mini gel electrophoresis (Invitrogen Inc., Waltham, MA, USA) and transferred to a nitrocellulose membrane (Invitrogen Inc.). The membrane was then washed using Tris-buffered saline (TBS; 20 mM Tris base, 137 mM NaCl, pH 7.6) containing 0.1% Tween 20 (TTBS) and blocked in TTBS containing 5% BSA solution for 2 h. The membrane was incubated overnight at 4 °C with primary antibodies diluted in TTBS (1:1000). Primary antibodies against p-JNK, JNK, p-ERK, ERK, p-p38, p38, p-IκB-α, IκB-α, p-STAT, and STAT were used. The membranes were washed four times with TTBS. Each membrane was incubated with secondary peroxidase-conjugated goat immunoglobulin G (IgG; 1:5000) for 1 h and washed five times with TTBS. Target proteins were detected using an enhanced chemiluminescence (ECL) solution. Images were captured using a ChemiDoc (Bio-Rad). For quantitative analysis, protein expression was determined using the Image Lab software (version 5.2.1; Bio-Rad).

### 3.8. UPLC-MS/MS Analysis

An Exion UPLC and SCIRX QTRAP 4500 mass spectrometer equipped with an ESI interface was used (SCIEX, Framingham, MA, USA). The Luna Omega polar C18 column (150 × 2.1 mm, 1.6 μm, Phenomenex, Torrance, CA, USA) was employed for chromatographic separation at 35 °C. The mobile phase consisted of 0.1% formic acid (A) and acetonitrile (B) and the flow rate was 0.25 mL/min. Gradient elution was performed as follows: 0–1 min, 10% B; 1–16 min, 10–95% B; 16–19 min, 95% B. Nitrogen (N_2_) was used as the collision gas. The ion spray voltage was 5.5 kV, and the temperatures were 600 °C. The declustering potential(DP) voltage and collision energy value were 30 and 20.

### 3.9. Data Analysis

All data are expressed as the mean ± standard deviation of at least one replicate. Student’s *t*-tests and one-way analysis of variance (ANOVA) were used for statistical analyses and statistical significance was set at *p* < 0.05.

## 4. Discussion

AD-like skin disease is a multifactorial skin disease with a complex relationship between innate and adaptive immune responses as well as environmental, genetic, and psychological factors [25]. AD is caused by a combination of severe pruritus, epidermal barrier abnormalities, imbalanced immune responses, and genetic predisposition [26]. From an immunological perspective, AD is a helper (Th)-mediated skin disease. This is caused by an imbalance between the Th1 and Th2 cells [27]. Some inflammatory cytokines and chemokines secreted by Th2 cells such as keratinocytes and mast cells can affect skin cells. Drugs, such as corticosteroids, calcineurin inhibitors, and antihistamines, which are currently used to treat AD, cause serious side effects with long-term use. Therefore, there is a need to develop natural plant-derived drugs with high efficacy and minimal adverse effects for the treatment of AD.

AD is associated with keratinocytes and immune cells. Therefore, we examined the anti-inflammatory effects and underlying molecular mechanisms of RCE in mouse RAW 264.7 macrophage and human keratinocyte HaCaT cells.

Inflammation plays an important role in the pathogenesis of several diseases. Inflammation is crucial for the defense against microbial infections. When bacteria and other pathogens enter our body, macrophages secrete inflammatory mediators including NO, PGE_2_, and proinflammatory cytokines (IL-6, IL-1β, and TNF-α) that attract immune cells to the site of infection and activate cells to eliminate them. And macrophages are involved in the early response to LPS; LPS stimulates toll-like receptor 4 (TLR4) to activate transcription factor nuclear factor-κB (NF-κB) and mitogen-activated protein kinases (MAPKs) pathways, which is the key molecule in inflammatory responses [28]. Therefore, we evaluated the anti-inflammatory effects of RCE by measuring the levels of inflammation-related factors in RAW 264.7 cells. We confirmed that the RCE concentrations of 50, 100, and 200 g/mL did not show cytotoxic effects against RAW 264.7 and used these concentrations for subsequent experiments. Next, we found that RCE inhibited the actions of various inflammatory mediators via NO, PGE_2_, IL-6, IL-1β, and TNF-α_,_ which are downstream signaling factors of COX-2, in a dose-dependent manner.

Keratinocytes are a major group of epidermal cells that play a key role in the pathogenesis of inflammatory skin lesions. When keratinocytes are damaged by repetitive mechanical stimulation, such as scratching, various keratinocyte-derived cytokines and chemokines are secreted to promote inflammatory skin disease progression [29]. Previous studies have shown that the stimulation of keratinocytes by TNF-α and IFN-γ can trigger the expression of inflammatory mediators such as cytokines (IL-1β, IL-6, and TNF-α), chemokines (MCP-1, RANTES, TARC, MDC, CTSS, and CXCL8), and adhesion molecules, leading to skin inflammation [30].

In particular, MDC/CCL22 and TARC/CCL17 may play important roles in increasing the incidence of certain skin diseases, including AD. Previous studies have reported that serum concentrations of MDC/CCL22 and TARC/CCL17 are positively correlated with disease severity in patients with AD [31]. Considering these factors, MDC/CCL22 and TARC/CCL17 may be involved in the pathogenesis of AD. RANTES/CCL5 is predominantly chemotactic and activates T-cells in chronic inflammatory conditions, including cutaneous diseases such as atopic dermatitis and psoriasis [32]. CTSS is a lysosomal cysteine protease that functions as an elastase over a broad pH range in alveolar macrophages and may participate in the degradation of antigenic proteins to peptides for presentation to major histocompatibility complex (MHC) class II molecules. Additionally, CTSS is associated with inflammatory processes, including atherosclerosis and asthma, and plays a crucial role in inflammatory skin diseases such as AD by activating the protease-activated receptor 2 (PAR2) [33], which is involved in pain and itching signaling [34]. Therefore, the inhibition of the secretion of proinflammatory chemokines and cytokines may be a potential therapeutic strategy for AD. In this experiment, we elucidated that RCE inhibits the production of proinflammatory cytokines and chemokines, such as IL-1β, IL-6, TNF-α, TARC, MDC, RANTES, and CTSS in TNF-α- and IFN-γ-stimulated keratinocytes.

NF-κB is critical to the innate/adaptive immune response and inflammatory response, especially the Th1 response, and regulates inflammation caused by Th2 cell differentiation and activation [35]. The NF-κB family includes critical transcription factors that are activated by various stimuli, including TNF-α, IFN-γ, and LPS. Upon stimulation, NF-κB complexes in the cytoplasm translocate to the nucleus, where they participate in the expression of numerous proinflammatory genes [36,37]. NF-κB signaling pathways are involved in the regulation of proinflammatory cytokines, such as IL-6, IL-8, and IL-1β, and chemokines, including TARC and MDC in HaCaT cells [38]. The STAT1 signaling pathway is one of the main inflammatory signaling pathways activated by several inflammatory cytokines such as interleukins and interferons [39,40]. STAT1 activation is known to be regulated by the activation of JAK. IFN-γ phosphorylates STAT1 via JAK1/2. Phosphorylated STAT1 increases TARC expression [41]. Thus, the inhibition of NF-κB and STAT1 activation plays a key anti-inflammatory role in AD. We evaluated whether RCE efficacy was mediated by the downregulation of NF-κB and STAT1 activation. The TNF-α- and IFN-γ-induced phosphorylation of NF-κB/IκBα and degradation of IκBα were noted in the HaCaT cells treated with RCE, indicating that RCE can affect the NF-κB signaling pathway. RCE significantly downregulated the phosphorylation of STAT1. These findings suggest that RCE significantly inhibited the expression of proinflammatory cytokines and chemokines by blocking NF-κB and STAT1 activation in IFN-γ- and TNF-α-treated HaCaT cells.

MAPK are serine/threonine-specific protein kinases that respond to external stressors. MAPK signaling pathways are involved in several cellular processes, including gene expression, cell proliferation, cell death, and survival [42]. Treatment with TNF-α and IFN-γ activates major MAPK factors such as p38, JNK, and ERK in HaCaT cells [43]. MAPK inhibition reduces intracellular signaling pathways and inflammatory cytokines [44]. We evaluated whether the efficacy of RCE was mediated by downregulation of MAPK activation. TNF-α- and IFN-γ-induced phosphorylation of p38, JNK, and ERK increased, but that of p38, and JNK decreased following RCE treatment. Thus, RCE treatment blocked TNF-α- and IFN-γ-induced production of inflammatory cytokines and chemokine by inhibiting p38 and JNK phosphorylation. Moreover, we aimed to identify the precise MAPK signaling pathway involved in RCE inhibition of TNF-α- and IFN-γ-induced production of proinflammatory cytokines (TNF-α, IL-6) and chemokines (MDC/CCL22 and RANTES/CCL5) in HaCaT cells, utilizing specific kinase inhibitors for ERK(PD98059), JNK(SP600125), and p38(SB203580). Phosphorylation and activation of each gene were significantly suppressed when the inhibitor and RCE were used in parallel. This indicates that a synergistic effect occurs when the inhibitor and RCE are administered in parallel, rather than when they are administered individually.

Increased levels of proinflammatory cytokines and chemokines cause alterations in skin barrier proteins (e.g., filaggrin, involucrin, hyaluronic acid, and aquaporins), which are some of the main initial factors in the pathogenesis of AD. Some proteins, such as filaggrin and involucrin, which play major roles in keratinocyte differentiation and skin-barrier function, resist the cell surface [45]. Mutations in filaggrin have been reported to be the main cause of AD, asthma, and allergic rhinitis [46]. In addition, hyaluronic acid is a major extracellular matrix component that plays important roles in physiological progress, such as skin moisturizing, antiaging, anti-inflammation, skin repair, and wound recovery [47]. Aquaporin is a small hydrophobic integral membrane protein that regulates the water retention rate in the skin and other organs. Aquaporin is a water- and glycerol-carrying protein produced by keratinocytes that is involved in their movement and differentiation. It plays an important role in restoring skin-barrier function [48]. Therefore, we examined the effects of RCE on the expression of skin-barrier proteins in HaCaT cells by ELISA. RCE significantly induced the expression pf filaggrin, hyaluronic acid, and aquaporin, which play important roles in the physical-barrier and water-retention properties of the skin, in HaCaT cells.

Finally, we investigated which compounds play important roles in the inflammatory effects of RCE. The UPLC-MS/MS results revealed two major flavonoids as rutin and kaempferol 3-*O*-rutinoside. Rutin has several attractive features as a drug such as its natural source, safety, cost-effectiveness, and wide spectrum of pharmacological actions [49,50]. Moreover, several reports have shown rutin to have antioxidant, antidiabetic, anticancer, anti-inflammatory, antibacterial, antiarthritic, and neuroprotection activities. Kaempferol 3-*O*-rutinoside is known for its anti-inflammatory activity through the NF-κB and MAPK pathways [51]. Therefore, we anticipate that rutin and kaempferol 3-*O*-rutinoside, components contained in RCE, are helpful in alleviating atopic dermatitis-like skin symptoms.

In this study, we confirmed that RCE suppresses the expression of AD-related cytokines and chemokines in TNF-α and IFN-γ-induced HaCaT cells. These effects are considered to be associated with the suppression of activation of the upstream NF-κB, STAT, and MAPK signaling pathways. Moreover, RCE significantly increased the expression of the skin components. These results provide a scientific basis for the use of RCE in the treatment of AD. However, additional research is needed to identify the bioactive ingredient that exhibits anti-inflammatory and antiatopic effects in RCE and to investigate efficacy in AD animal models. In addition, comparative verification studies with drugs used in actual treatment must be necessarily accompanied. Although further research is needed, our data suggest that RCE is a potential therapeutic drug candidate for the treatment of inflammatory skin diseases.

## 5. Conclusions

Our experimental finding provide adequate scientific evidence for use of RCE in the treatment of inflammatory diseases. We demonstrated for the first time that RCE exerts anti-atopy activates by suppressing TNF-α/IFN-γ-induced expression of cytokines (TNF-α, IL-1β, and IL-6) and Th2 chemokines (TARC, MDC, RANTES, and CTSS) via blockade of the activation of the NF-κB, STAT1 and MAPK(JNK, p38) pathways in HaCaT cells. In addition, RCE significantly inhibited the expression of inflammatory mediators (NO, PGE_2_ TNF-α, IL-6, and COX-2) in RAW 264.7 cells (Supplementary Materials). Moreover, RCE increased the expression of filaggrin, hyaluronic acid, and aquaporins, which play an important role in the physical barrier of the skin. Although further research is needed, our results suggest that saponarin is a valuable candidate for alleviating inflammation and AD.

## Data Availability

The data are contained within this article.

## Supplementary Materials

The following supporting information can be downloaded at: https://www.mdpi.com/article/10.3390/molecules28145463/s1. Figure S1: Western blot analysis of COX-2 protein in RAW 264.7 cells treated with RCE; Figure S2: Western blot analysis of Ikb-α, p-Ikb-α, p-STAT, STAT protein in HaCaT cells treated with RCE; Figure S3: Western blot analysis of p-JNK, JNK, p-p38, p38, p-ERK, and ERK protein in HaCaT cells with RCE.
