# Supplementary material for: Anti-Inflammatory and Antiatopic Effects of Rorippa cantoniensis (Lour.) Ohwi in RAW 264.7 and HaCaT Cells
Source: Molecules. 2023 Jul 17;28(14):5463. doi: 10.3390/molecules28145463 (PMC10383606; doi:10.3390/molecules28145463)
Supplement: Supplementary file 1 [file molecules-28-05463-s001.zip › molecules-2441091-supplementary.pdf]

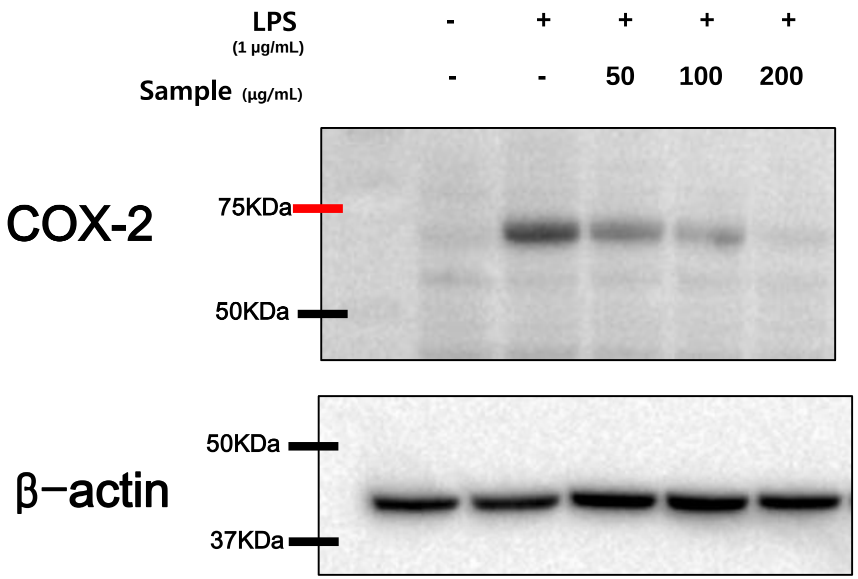


**Figure S1. Original western blot for Figure 2 showing the bands with molecular weight markers.**

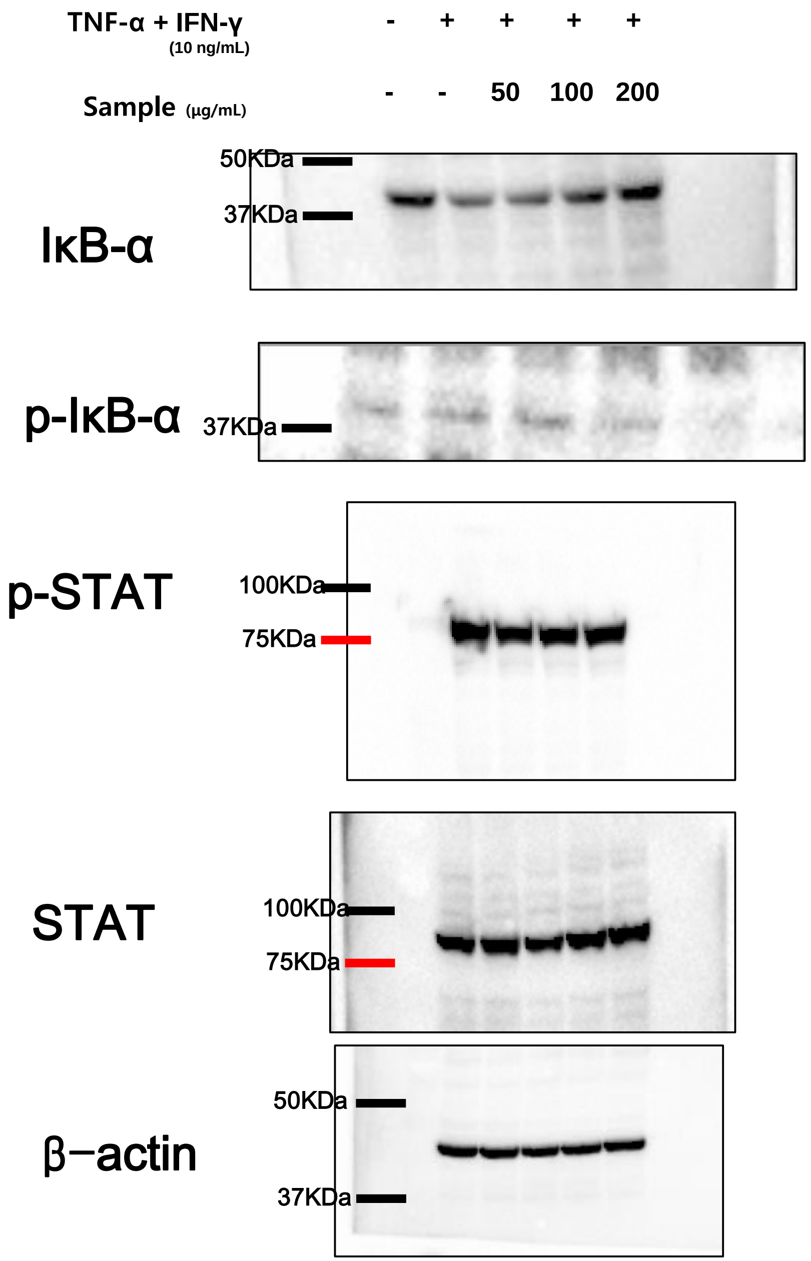


**Figure S2. Original western blot for Figure 4 showing the bands with molecular weight markers.**

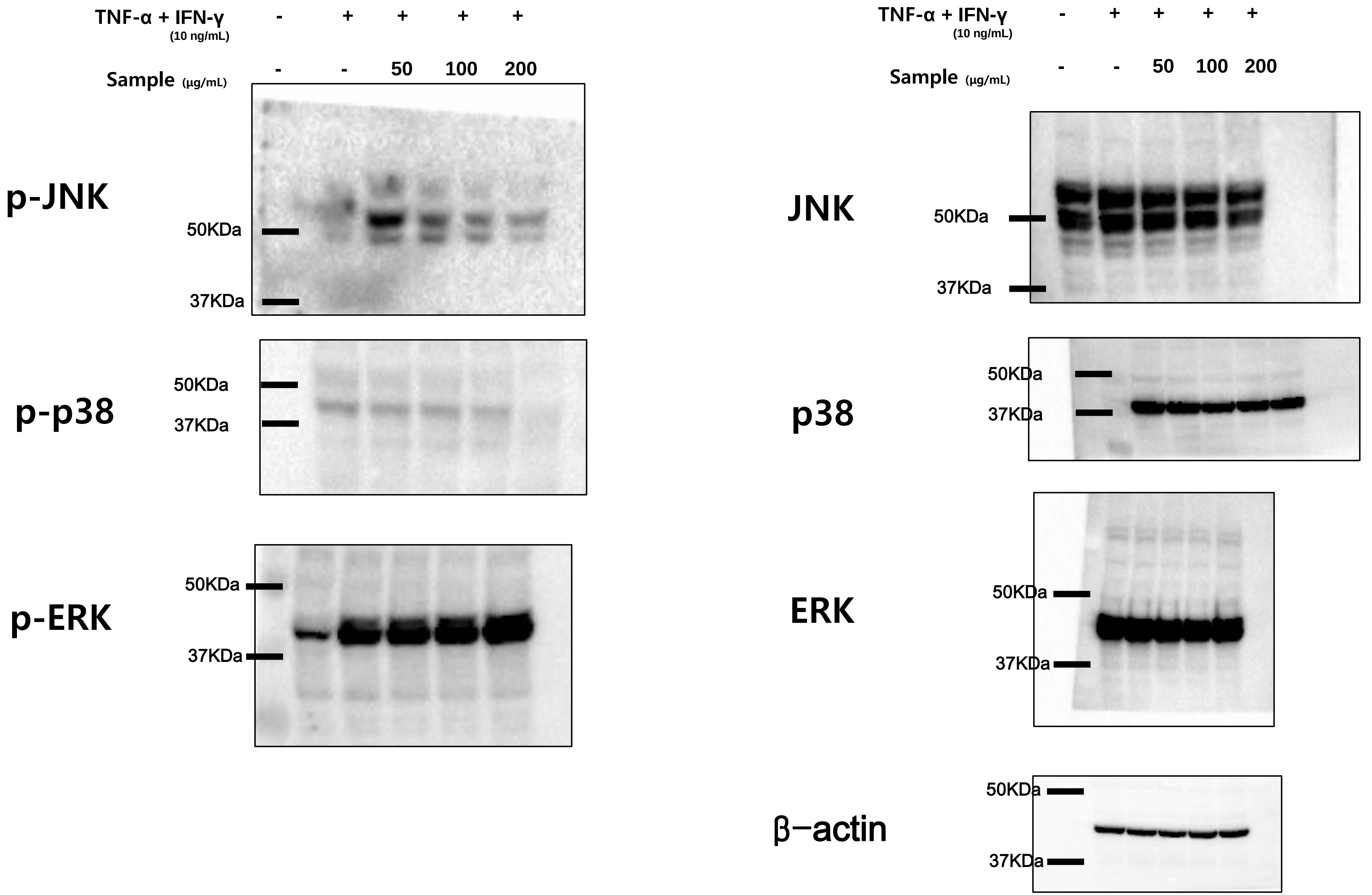


**Figure S3. Original western blot for Figure 5 showing the bands with molecular weight markers.**
